# Perceived stress and salivary biomarkers in educators: comparison among three stress reduction activities

**DOI:** 10.1080/21642850.2022.2102016

**Published:** 2022-07-22

**Authors:** Doreen Wagner, Sharon M. Pearcey

**Affiliations:** a WellStar School of Nursing, Kennesaw State University, Kennesaw, GA, USA; b Department of Psychological Science, Kennesaw State University, Kennesaw, GA, USA

**Keywords:** Cortisol, meditation, stress reduction, teacher stress, yoga

## Abstract

**Background:**

The teaching profession is a potentially stressful occupation with up to 30% of all novice teachers leaving the profession and annual teacher turnover is higher when compared with turnover of all other occupations. This study investigated the effects of a one-time stress reduction activity (meditation, yoga, or aerobic exercise) in university and K-12 educators who were part of one-day seminar on Stress Reduction.

**Methods:**

Participants (N = 26) self-selected their stress reduction activity, completed a demographic questionnaire, educator stress self-assessment tool, and visual analogue scales indicating current stress levels. Salivary cortisol and amylase levels were measured before, immediately after, and 30 minutes after completion of the stress reduction activity.

**Results:**

Three (time) by three (activity) mixed factorial ANOVAs were computed for salivary analytes. The ANOVA for cortisol revealed a significant interaction (F (4, 66) = 3.60, *p* = .01). Comparisons showed significant differences with the aerobic exercise group having significantly higher cortisol levels at the 30-minute post-activity level when compared to the meditation (*p* < .05, Cohen’s d = .74) and yoga groups (*p* < .05, Cohen’s d = .52).

**Conclusion:**

Overall, the one-time activity of meditation and yoga showed lowered salivary cortisol levels at 30-minutes post-activity when compared to aerobic exercise activity. Additional research to examine the effects of stress reduction on educators in the work setting is needed.

## Introduction

It is estimated that US employers lose approximately 84 billion dollars a year from loss of productivity due to absenteeism (Witters & Liu, 2013). With up to 10% of all unscheduled absences attributed to job stress, calling in stressed or taking a ‘mental health’ day is one of the most common non-illness related reasons for job absenteeism (Health Care Collector, 2017). Specifically, job stress occurs when there is a disconnect between job requirements and capabilities, resources, or needs of the worker (National Institute for Occupational Safety and Health, 1999).

The teaching profession is not immune from job stress, in fact, quite the contrary. Before the COVID-19 pandemic, literature suggests that teacher burnout is a chronic problem leading to high teacher attrition rates (Rumschlag, 2017). Inexperienced teachers have an extremely high attrition rate with national estimates ranging from 19% to 30% during their first 5 years of teaching (Sutcher, Darling-Hammond, & Carver-Thomas, 2016). There are numerous reasons for this attrition; inherent demands of teaching, high levels of stress, and burnout are foremost (Sas, Boros, & Bonchis, 2011; Sutcher et al., 2016). Prior to the pandemic, one in six teachers said they were likely to leave teaching whereas by the end of the 2020–21 school year, almost one in four teachers were likely to leave their job (Steiner & Woo, 2021).

In a survey of over 30,000 K-12 educational personnel of which 80% were teachers (American Federation of Teachers and the Badass Teachers Association, 2015), 73% of the participants reported that they ‘often’ found their work stressful, whereas only 2% reported that their work was ‘rarely’ stressful. Stress takes a toll on health outcomes with 26% of these respondents indicating that of the last 30 days, their mental health, as defined by stress, depression, and emotional challenges, was not good for at least 9 or more days. In a more recent survey, K-12 teachers reported depression at a rate 2 ½ times more than the general adult population (Steiner & Woo, 2021).

Teaching is unique compared to other means of employment. With this distinctive position, comes unique stressors even before the stress of the pandemic. In a nationwide survey of K-12 teachers in the US, the top five sources of stress reported by teachers are the needs of students, too many work-related duties and responsibilities, lack of control regarding decision making, unmotivated students, and the pressure of being ‘accountable’ (Richards, 2012). Other sources of stress reported by K-12 teachers and other educational personnel are a lack of autonomy, time pressures, student discipline problems, lack of training or professional development, long hours, lack of administrative support, and a widening pay gap when compared to occupations with similar education levels (Allegretto & Mishel, 2016; McCarthy, 2019; Skaalvik & Skaalvik, 2011; Steinhardt, Smith Jaggars, Faulk, & Gloria, 2011). Since the pandemic, most K-12 teachers stated the stress of teaching was the chief cause for leaving their teaching job. In addition, teachers named inadequate pay not worth the health risks of teaching during COVID-19 (Steiner & Woo, 2021).

Similarly, college and university faculty experience high levels of occupational stress from teaching workload, research responsibilities, demands to publish, and the 24-hour demands of on-line technology (McNaughton-Cassill, 2017; Shin & Jung, 2014). COVID-19 caused faculty researchers more stress due to the negative impact of halted or delayed research and closed laboratories (Carr et al., 2021). On average, faculty lost up to 24% of research productivity, with laboratory-based female scientists who had young, dependent children losing the most research time when compared to pre-pandemic levels (Myers et al., 2020).

Faculty retention is problematic for universities. A longitudinal study following 2,966 assistant professors at 14 universities in the United States found that the median time a faculty member stays at a university is 10.9 years (Kaminski & Geisler, 2012). Job satisfaction, intent to leave, as well as emotional and physical health has been related to the institution climate and support of work-life integration policies (Kaminski & Geisler, 2012). Findings from a study of non-tenure track faculty show workload, instability of status, lack of physical support, and pay inequity as workplace stressors (Reevy & Deason, 2014). In a nationwide survey of 16,112 undergraduate teaching faculty members at 269 colleges and universities, only 37.5% of respondents felt they attain a healthy work-life balance ‘to a great extent’ and found lack of personal time, teaching load, committee work, and colleagues as the highest sources of stress related to their career (Eagan et al., 2014). In a recent systematic review of faculty burnout from continued occupational stress, authors found age had a negative relationship with burnout, no correlation between years of experience and burnout in all but two studies, and mixed findings related to the role of rank in burnout (Sabagh, Hall, & Saroyan, 2018).

High levels of workplace stress not only affect job satisfaction and retention but may also affect the health of educators. The stress response activates two major physiologic axes in the autonomic nervous system: the sympathetic-adrenomedullary (SAM) axis and the hypothalamic–pituitary–adrenal (HPA) axis. Cortisol is a stress hormone released by the HPA during times of stress. Salivary α-amylase (sAA) is a correlate of sympathetic activity representing physiological and psychological stress that yields a rapid response to a stressor and a subsequent rapid recovery (Nater & Rohleder, 2009). It is generally accepted that chronic stress stems from repeated exposure to situations that lead to the release of stress hormones like adrenaline and cortisol. This type of continual stress leads to the dysregulation of the stress response system and may lead to negative health outcomes such as heart disease, diabetes, depression and decreased immune response (McEwen, 1998).

A study on immune function and stress hormone activation in faculty members showed significant increases in immune activation (Tumor Necrosis Factor, Interleukin-2 and Interleukin-4) with increased concentrations of salivary cortisol and amylase on lecture days in comparison to non-lecture days (Filaire et al., 2011). Additionally, middle school teachers have been shown to have blunted cortisol awakening response, indicative of chronic stress, at the midpoint of the academic year when compared to the beginning of the year indicating a higher stress response that has accumulated over the year (Katz, Harris, Abenavoli, Greenberg, & Jennings, 2018). In a systematic review and meta-analysis of workplace stress, greater workplace stress was associated with lowered immunity than over-commitment and that over-commitment moderated the relationship between immunity and workplace stress; therefore, one’s perceptions of stress may allow individuals to be more open to stress-reduction interventions (Eddy, Heckenberg, Wertheim, Kent, & Wright, 2016).

Efforts to reduce stress levels at work may help educators counteract chronic stress at the workplace and ultimately affect health outcomes. Perception of stress is individualized and ultimately impacts physiological and mental health. Stress-reduction activities such as yoga, meditation, and aerobic exercise are commonly associated with beneficial effects on health, and evidence supports these activities for reducing educator stress (von der Embse, Ryan, Gibbs, & Mankin, 2019).

Yoga is a combination of spiritual and mind–body activities that facilitate balance and calmness using breathing, meditation, and physical postures in varied styles (Cramer et al., 2016a). A systematic review of the health benefits of various yoga styles indicated that in more than 90% of randomized control trials, individuals who engaged in yoga had positive health outcomes (Cramer, Lauche, Langhorst, & Dobos, 2016b).

Meditation is a form of mental training practice and is frequently categorized into three types; devotional (focusing on understanding religious or spiritual meaning), mantra-based (focusing on a word or phrase), and mindfulness-based (focusing on the present) (Burke, Lam, Stussman, & Yang, 2017). A recent study indicates that 11.9 million American adults have reported practicing meditation in their lifetime with wellness and prevention as the most likely reason for this practice (Burke, et al.). Meditation, in general, has been shown to have health benefits including lowered blood pressure, reduced blood glucose level following a meal in diabetics, decreases in chronic pain, and psychological benefits (Horowitz, 2010).

The relationship among exercise and both physical and mental health has been demonstrated in a multitude of ways. Aerobic activity is associated with improved vascular fitness, decreased hypertension (Roque et al., 2013) and increased insulin sensitivity that impacts Type-2 diabetes risk (Yaribeygi, Atkin, Simental-Mendia, & Sahebkar, 2019). It decreases stress, increases sleep quality, and increases overall perception of well-being (Gerber et al., 2013; Wang & Boros, 2021). Exercise also has a role in decreasing anxiety symptoms in clinical populations through physiological mechanisms including decreasing oxidative stress and inflammation (Moylan et al., 2013).

Although the positive relationship between aerobic exercise and overall health is evident, the acute effects of aerobic exercise are physiologically stressful due to the increase in sympathetic nervous system (SNS) activity. The differential SNS effects of exercise to acute bouts of strenuous exercise, when compared to routine exercise regimes, must be considered when describing the relationship among exercise and perceived and physiological stress.

The teaching profession is a stressful occupation. The use of stress reduction activities has been shown to reduce stress levels and may help educators decrease the impact of workplace stress on their health (von der Embse et al., 2019). This research explored three common activities as possible stress reduction approaches for educators. Therefore, the purpose of this study was to investigate perceived stress in University and K-12 educators and to determine whether one occurrence of a stress-reduction activity (yoga, meditation, or cardiovascular exercise) could reduce perceived stress and have an effect on cortisol and amylase responses.

## Materials and methods

The study was approved by the university Institutional Review Board (#13-235) and informed consent was obtained prior to participation. No incentives were given. The study group consisted of 26 adult educators (*M* age = 38.8; range = 26–64; 61% Female). All participants were full-time teachers with an average of 10.81 (*SD *= 6.21) years teaching experience. The sample was somewhat racially diverse with 53.8% White, 30.8% Black, 7.7% other, and 7.7% chose not to answer. The participants reported exercising an average of 2.42 (*SD* = 1.64) days per week and 49.04 (*SD *= 46.28) minutes each time they exercised. Participant weight and height demographics can be seen in Table 1.

**Table 1.** Weight and height characteristics of male and female participants.

Females (N = 16)	Mean (sd)	Range
Height (cm)	164.05 (6.10)	157.5–180.3
Weight (kgs)	81.19 (17.46)	53.52–108.86
Males (N = 10)		
Height (cm)	179.83 (12.14)	152.4–198.1
Weight (kgs)	92.40 (21.48)	45.36–127.01

**Figure 1.**
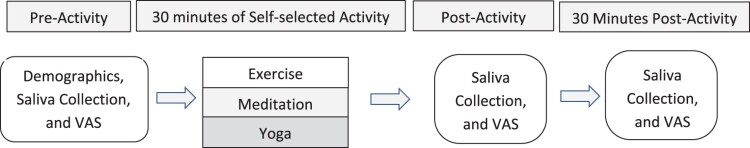
Study design protocol for stress reduction activity.

Upon arrival, participants self-selected activities in which to participate. Eleven participants chose yoga, nine chose meditation, and six chose an aerobic exercise workout. Each participant engaged in 30 minutes of their chosen activity which was led by a professional instructor. Stress levels and saliva samples were taken before participants engaged in their chosen stress reduction activity (pre-activity), immediately after the activity (post-activity), and 30-minutes post-activity. Participants indicated their current stress level on 100 mm visual analogue scales (VAS) with the anchors ‘No Stress’ and ‘High Stress’. Figure 1 contains the study design model. Saliva samples were collected by the passive drool technique and assayed using commercially available kits from Salimetrics (State College, PA). To avoid contamination, participants were instructed to avoid eating or drinking, excessive exercising, and smoking one-hour before saliva sampling. No saliva flow stimulants were used.

Whole saliva specimens using a passive drool method allows for the collection of large sample volumes (1–5 mL), multiple assays of each sample, and minimizes threats to assay accuracy and validity (Granger et al., 2007). To minimize known diurnal variations in hormone levels, saliva samples were collected at approximately the same time of day for each sampling occurrence (9:00 am to 12:00 pm). Samples were frozen at −80°C until assayed. Frozen samples were thawed at room temperature and centrifuged to separate any cell debris and mucins prior to assay.

High correlations between serum and salivary cortisol have been reported throughout the physiological concentration range in many typical samples (i.e. men, women, pregnant women) and under experimental manipulation (i.e. cortisol sodium succinate injection and adrenocorticotropic hormone administration), indicating that salivary cortisol levels reliably estimate serum cortisol levels (Vining, McGinley, Maksvytis, & Ho, 1983).

Cortisol assays were prepared using high sensitivity salivary competitive immunoassay kits (Salimetrics, State College, PA) to determine free cortisol concentrations in saliva samples. The range of standards was 0.012 µg/dL to 3.000 µg/dL. Each assay was completed in duplicate using 25 µL of saliva for each well and samples were re-assayed if the coefficient of variation between the duplicates was greater than 15%, unless the absolute value was less than 0.030 µg/dL. The average intra-assay coefficient of variation was 6.17%.

sAA is quantified using a kinetic reaction assay where α-amylase reacts to the chromogenic substrate, 2-cholor-p-nitophenol bound to maltotriose, yielding 2-cholor-p-nitophenol. Salivary samples are measured spectrophotometrically at 405 nm at 1 and 3 minutes. The difference value represents an increase in absorbance directly proportional to the sAA in each sample. Samples that exceeded 400 U/mL or were less than 2.0 U/mL were reassayed using varying dilutions to ensure accurate values (see Granger et al., 2006 for more detail).

## Results

**Figure 2.**
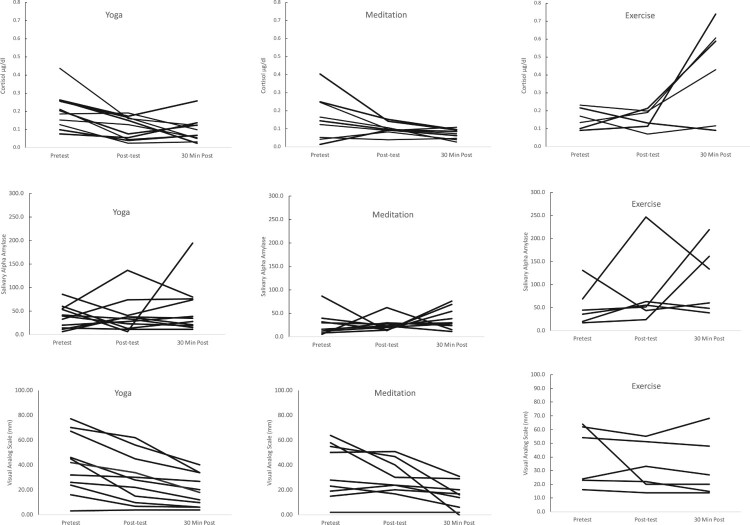
Spaghetti plots of raw data for each participant by group and activity.

Data were analyzed using two-way analysis of variance with repeated measures by using the General Linear Models Procedure in SPSS (Version 27.0) for Windows (IBM Corporation, 2020). The primary assumptions underlying the analyses, such as normality of the data and constant variance of error terms, were violated and necessary transformations of the data were performed to meet assumptions. Specifically, cortisol and sAA measurements were log-transformed and VAS scores were square root transformed to normalize the data. All statistical analyses were performed on transformed data; however, data table and figures are non-transformed for conceptual ease. Pre-activity measurements served as a baseline and there were no significant differences in cortisol, sAA, or VAS measurements among the participants of different activity groups at baseline. Plots of cortisol, sAA, and VAS values for each participant at each time period can be seen in Figure 2.

A 3 (Activity) by 3 (Time) mixed factorial ANOVA for VAS revealed a significant main effect for Time, *F*(1.44, 30.72) = 15.67, *p* = 0.000, η_G_^2 ^= .11 (corrected for sphericity violation using Greenhouse-Geisser correction). Fisher’s least significant difference (LSD) post hoc tests revealed that the mean VAS at pre-activity (*M* = 5.90, *SD* = 1.99) was significantly higher than the mean VAS at post-activity (*M* = 5.15, *SD* = 1.72; *M*_dif _= 0.72, *p* = .003, Cohen’s *d* = .41) and at 30-minutes post-activity (*M* = 4.19, *SD* = 1.80; *M*_dif_ = 1.58, *p* = .000, Cohen’s *d* = .90). In addition, the VAS at post-activity was significantly higher than the VAS at 30-minutes post-activity (*M*_dif _= 0.87, *p* = .003, Cohen’s *d* = .54). There was no significant main effect for activity or a significant interaction.

A 3 (Activity) by 3 (Time) mixed factorial ANOVA for cortisol revealed a significant main effect for Activity, *F*(2, 23) = 5.09, *p* = .015, η_G_^2 ^= .18. Fisher’s LSD post hoc tests indicated that the cortisol level of the exercise group (*M* = −1.66, *SD* = 0.19) was significantly higher than both the meditation group (*M* = −2.44, *SD* = 0.16; *M*_dif _= 0.78, *p* = .005, Cohen’s *d* = 1.10) and the yoga group (*M* = −2.23, *SD* = 0.14; *M*_dif _= 0.57, *p* = 03, Cohen’s *d = .78*). There were no differences in cortisol levels between the meditation and yoga groups.

**Figure 3.**
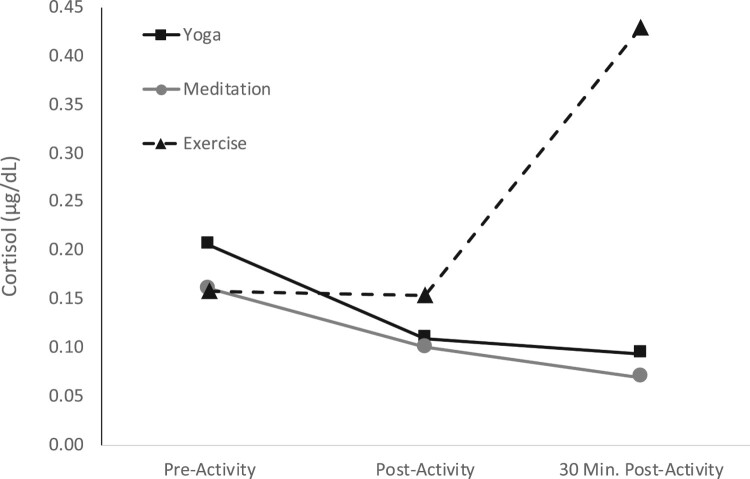
Cortisol (µg/dL) measured at pre-, post-, and 30 minutes post-activity for each activity group.

More notably, a significant interaction effect between Activity and Time was found for cortisol, *F*(3.86, 44.36) = 4.89, *p* = 0.003, η_G_^2 ^= .18 (corrected for sphericity violation using Huynh-Feldt correction). At pre-activity and post-activity, there were no significant differences in cortisol levels among the three activity groups. However, based on the Fisher LSD post hoc tests, at 30-minutes post-activity, the exercise group (*M* = −0.49, *SD* = 0.40) was significantly higher than the yoga group (*M* = −1.12, *SD* = 0.30; *M*_dif _= 0.63, *p* = .000, Cohen’s *d* = 1.87) and the meditation group (*M* = −1.19, *SD* = 0.21; *M*_dif _= 0.70, *p* = .000, Cohen’s *d = *.28). See Figure 3.

There was no significant main effect for Time, indicating there were no differences in cortisol levels at each time period for all activities combined. However, when looking at time differences for each activity individually, there were significant simple main effects. The cortisol levels of the yoga group dropped significantly from pre-activity to post-activity (*M*_dif_ = 0.72, *p* = .002, Cohen’s *d *= 1.16) and from pre-activity to 30-minutes post-activity (*M*_dif_ = 0.89, *p* = .006, Cohen’s *d *= 1.47). The cortisol levels of the exercise group increased significantly from post-activity to 30-minutes post-activity (*M*_dif_ = 0.82, *p* = .011, Cohen’s *d *= 1.15). There were no differences between the other time periods for cortisol. There were no differences in cortisol across time for the meditation group.

A 3 (Activity) by 3 (Time) mixed factorial ANOVA for sAA revealed a significant main effect for Activity, *F*(2, 23) = 6.75, *p *= 0.005, η_G_^2 ^= .19. Fisher LSD post hoc tests showed that the exercise group had significantly higher sAA levels (*M* = 1.78, *SD* = 0.34) than the yoga (*M* = 1.50, *SD* = 0.36; *M*_dif _= 0.28, *p* = .014, Cohen’s *d *= .80) and the meditation groups (*M* = 1.38, *SD* = 0.30; *M*_dif _= 0.40, *p* = .001, Cohen’s *d *= 1.28).

The mixed factorial ANOVA for sAA also revealed a main effect for Time, *F*(2, 46) = 3.03, *p *= 0.058, η_G_^2 ^= 0.07 (corrected for sphericity violation using Huynh-Feldt correction). However, based on the Tukey’s HSD post hoc tests, at 30-minutes post-activity (*M* = 1.64, *SD* = 0.36), sAA levels were significantly higher when compared to pre-activity (*M* = 1.44, *SD* = .37; *M*_dif _= .22, *p* = 0.009, Cohen’s *d *= .55). The post-activity group (*M* = 1.50, *SD* = .35) did not differ from the other two groups. No interaction effect was found and no significant effects for Time for each activity individually were found in sAA results.

## Discussion

There were interesting and statistically significant findings in this small, convenience sample of educators participating in one of three stress-reduction activities. The self-perception of stress was lowered similarly in all three activity groups with the lowest ratings at 30-minutes post-activity. Interestingly, the mean salivary cortisol level was significantly affected by the interaction of activity type and measurement time although no significant interaction effect related to any type of activity on sAA levels. Although our sample sizes were quite small, the effect sizes, represented by generalized η^2^ (η_G_^2)^ and Cohen’s *d* were of note. Specifically, for cortisol and sAA, the η_G_^2^’s ranged from .18 to .19 which is in between the medium to large range (Bakeman, 2005; Lakens, 2003). Although these effect sizes are high, they must be interpreted with caution. Because of our low sample size, it is expected that these effect sizes may be inflated, and if replicated, effect sizes would likely be much smaller (Button et al., 2013).

Following is a discussion related to findings by activity.

### Yoga

Several studies have investigated the effects of yoga on perceived stress and stress hormones. Like the findings of García-Sesnich, Flores, Ríos, and Aravena (2017) we found a reduction in perceived stress after participation in yoga that lasted after the activity. García-Sesnich et al. found a significant reduction in cortisol immediately following a yoga session; however, no differences were found in sAA. Our findings for cortisol and sAA align with García-Sesnich et al.’s findings. For our yoga group, the cortisol levels dropped significantly from pre-activity to post-activity. Additionally, our cortisol levels remained significantly lower than pre-test at 30-minute post-activity, showing a brief, but sustained response. As with García-Sesnich et al.’s study, no differences were seen in sAA in our yoga group.

Although our findings immediately following one yoga session seem encouraging, the effects of participating in yoga regularly are less clear. Sieverdes and colleagues (2014) found no significant differences in bedtime and awakening curves in salivary cortisol and sAA levels after three months of yoga practiced twice per week. However, concerning the perception of stress, several studies have shown a decrease in overall perceived stress after yoga programs ranging from 7 to 16-weeks (Hewett, Pumpa, Smith, Fahey, & Cheema, 2018; Lindahl, Tilton, Eickholt, & Ferguson-Stegall, 2016) with perceived stress scores being lower with increased number of classes attended (Bilderbeck, Brazil, & Farias, 2015; Hewett et al., 2018).

### Meditation

A systematic review and meta-analysis on the effects of all types of meditation on stress reduction concluded that mindfulness-based meditation was associated with decreased self-reported measures of stress and biomarkers of stress reactivity in differing populations (Pasco, Thompson, Jenkins, & Ski, 2017). In our study, we had similar findings as there was a significant reduction in the VAS from pre-activity to 30-minutes post-activity and post-activity to 30-minutes post-activity.

Although meditation has been shown to decrease the subjective experience of stress, the literature on its relationship with cortisol is not as clear. In an eight-week mantra-based meditation study, participants had lower blood pressure and reduced perception of stress, however, no differences in cortisol were found between the groups (Chambers, Phillips, Burr, & Xiao, 2016). In a workplace study, researchers found meditation effective in reducing cortisol production, as indicated by lowered high and low diurnal cortisol slopes. However, no changes were found for cortisol response at single time-points or at awakening. In contrast, decreased cortisol, C-reactive protein, blood pressure, heart rate, triglycerides, and tumor necrosis factor-alpha were found in a systematic review and meta-analysis of mindfulness meditation practices (Pasco et al., 2017). In our cross-sectional study, we found no differences in cortisol across time for the meditation group. Interestingly, all studies that showed cortisol changes were long-term cortisol changes rather than short term changes.

Arch and colleagues (2014) showed meditation groups had a lower overall sAA response when compared to control groups. In a follow up study, it was concluded that the relationship between sAA and meditation is complex and moderated by other psychological traits (Arch, Landy, & Brown, 2016). In our repeated measures cross-sectional study, there were no significant effects for time related to sAA.

### Aerobic exercise

The relationship among exercise, physiological stress, and perceived stress is complex. When considering effects of general level of activity on basal cortisol levels, physically active men had lower daily AUCg (area under the curve with respect to ground) cortisol level when compared to sedentary men (Gubelmann, Vollenweider, & Marques-Vidal, 2018). More specifically, when comparing the sedentary, those who engage in high intensity activities mainly on weekends (weekend warriors), and individuals who regularly exercise, daily cortisol AUCg was lower for those who were regularly active with no differences found between the weekend warriors and the sedentary (Gubelmann et al., 2018). Contrastingly, Hayes et al. (2015) found no differences in resting cortisol between lifelong exercisers and lifelong sedentary males (Hayes et al., 2015).

The fluctuations in cortisol during an exercise challenge seems to be dependent on exercise intensity and general fitness level. For endurance trained males, cortisol levels increased significantly and remained elevated for 60 minutes post-exercise during interval training. However, when the same males engaged in tempo running and circuit training there was only a significant increase in salivary cortisol levels from resting state to immediately post-exercise with no differences between rest and 15-, 30-, or 60-minutes post-exercise (Tanner, Nielsen, & Allgrove, 2014). VanBruggen, Hackney, McMurray, and Ondrak (2011) only found increases in salivary cortisol levels from pre-test to post-test and 30-minutes post-test for high intensity exercise (60% VO2max), compared to low (20% VO2max) and moderate intensity (40% VO2max) exercise among endurance trained males. Comparing athletes to sedentary men, Koc (2018) found an increase in serum cortisol after a bout of exercise in elite male athletes, but no difference was found pre- and post-exercise in sedentary controls. In contrast, in non-athletes, cortisol levels increased during intense exercise using a treadmill test and remained significantly higher than resting levels up to 24 hours after completion of the exercise (Rahman, Abdullah, Singh, & Sosroseno, 2010).

Our findings show that cortisol was significantly higher in the aerobic exercise group compared to the yoga and meditation groups. This difference seemed to be driven by the increase in cortisol shown 30-minutes after exercising for the aerobic exercise group. Another study examining acute effects of one-time interventions of African dance and yoga showed significantly higher cortisol in the dance group when compared to the yoga group (West, Otte, Geher, Johnson, & Mohr, 2004). Consistent with the findings of VanBruggen et al. (2011), our study showed a significant increase in cortisol 30-minutes after engaging in a single bout of intense aerobic exercise. However, we did not assess cortisol levels more than the 30-minutes post-activity to see if levels remained elevated.

Considering perceived stress, we found no changes in reported stress in the aerobic exercise group throughout the study. However, as with previous findings, we found a disconnect between self-assessment (VAS) and physiological measurements (cortisol) of stress for the exercise group. As perceived stress remained constant, cortisol levels increased significantly 30-minutes post-activity. This disconnect is consistent with findings that perceptions of stress are not aligned with physiological measures of stress (Ursache, Noble, & Blair, 2015) and they may be moderated by other factors (Pulopulos & Kozusznik, 2018).

## Limitations

This study was limited by several factors. Small sample size with an unequal number of male and female participants restricts the interpretation and generalization of results. This was an applied seminar educating participants on stress reduction techniques. We chose not to include a control group to allow all participants the benefit of participation and because of the small number of participants available. Additionally, in order to make the seminar more naturalistic, we allowed participants to self-select their stress reduction activity. However, this led to uneven distribution of participants to type of group. This study only included one 30-minute activity and did not investigate multiple activity sessions. In future research, it would be important to gather biomarker measures on repeated activity sessions across time. Another limitation was that the study explored acute response to the three stress reduction activities and could not be generalized to long term responses.

We did not control for phase of menstrual cycle or use of oral contraceptives, which may have influenced cortisol levels. In addition, we did not have baseline awakening biomarker values to compare with the one-time activity participants chose. Lastly, the measures taken on the VAS and the stress questionnaire may have influenced salivary measures by bringing greater self-awareness to specific states.

## Conclusions

Overall, the activities of meditation and yoga reduced salivary cortisol levels and showed a continued decrease in salivary cortisol levels at 30-minutes post-activity. It appears that one-time meditation and yoga sessions activated more of a relaxation response through the parasympathetic nervous system and thus decreased cortisol. Meditation, as an intervention that could easily be done in the workplace, may be beneficial for educators to decrease stress levels and impact their biophysical as well as psychological being in a positive way. There is little research on the short-term effects of yoga on cortisol levels. However, there are health benefits to long-term yoga practice, including decreased blood pressure, cortisol levels, and anxiety. Implications of this study point to the need for further research to examine the positive effects of meditation and yoga on educators in the work setting. A more in-depth study including baseline values of awakening biomarker values is required to further investigate the effects of stress-reduction interventions in educators.

## Data accessibility

Data are not openly accessible because sharing of data was not stated in the consent form. Please contact S. Pearcey for questions about data.
